# CRP Interacts Specifically With Sxy to Activate Transcription in *Escherichia coli*

**DOI:** 10.3389/fmicb.2019.02053

**Published:** 2019-08-30

**Authors:** Emilie Søndberg, Anurag Kumar Sinha, Kenn Gerdes, Szabolcs Semsey

**Affiliations:** Centre for Bacterial Stress Response and Persistence, Department of Biology, University of Copenhagen, Copenhagen, Denmark

**Keywords:** CRP, Sxy, CRP-S, TfoX, SIR

## Abstract

Horizontal gene transfer through natural competence is an important driving force of bacterial evolution and antibiotic resistance development. In several Gram-negative pathogens natural competence is regulated by the concerted action of cAMP receptor protein (CRP) and the transcriptional co-regulator Sxy through a subset of CRP-binding sites (CRP-S sites) at genes encoding competence factors. Despite the wealth of knowledge on CRP’s structure and function it is not known how CRP and Sxy act together to activate transcription. In order to get an insight into the regulatory mechanism by which these two proteins activate gene expression, we performed a series of mutational analyses on CRP and Sxy. We found that CRP contains a previously uncharacterized region necessary for Sxy dependent induction of CRP-S sites, here named “Sxy Interacting Region” (SIR) encompassing residues Q194 and L196. Lost promoter induction in SIR mutants could be restored in the presence of specific complementary Sxy mutants, presenting evidence for a direct interaction of CRP and Sxy proteins in transcriptional activation. Moreover, we identified constitutive mutants of Sxy causing higher levels of CRP-S site promoter activation than wild-type Sxy. Both suppressor and constitutive mutations are located within the same area of Sxy.

## Introduction

The cAMP receptor protein (CRP; also known as the catabolite activator protein, CAP) is a versatile transcriptional regulator, regulating more than a hundred genes in *Escherichia coli.* Most of these genes are involved in transport and utilization of different carbon sources (Kolb et al., 1993; Zheng et al., 2004; Grainger et al., 2005). In the presence of the allosteric effector cyclic adenosine monophosphate (cAMP), CRP binds to specific symmetrical 16 bp DNA sites (5′-T_4_G_5_**T**_6_G_7_A_8_-6N-T_15_C_16_**A**_17_C_18_A_19_-3′) and regulates the activity of nearby promoters (Kolb et al., 1993). Depending on the topology of the promoter and regulatory sites, cAMP-CRP can either activate or repress transcription, using different molecular mechanisms (Aiba et al., 1981; Zheng et al., 2004; Hunziker et al., 2010; Lee and Busby, 2012).

The simplest cAMP-CRP activated promoters can be divided into two classes depending on the relative position of the DNA binding site and the consequent interactions between CRP and the RNA polymerase (RNAP) (Busby and Ebright, 1999). In Class I promoters, the cAMP-CRP binding site is located upstream of the core promoter sequence. Direct interaction between the activation region 1 (AR1) of the DNA-bound cAMP-CRP protein and the C-terminal domain of the RNAP α subunit (αCTD) facilitates RNAP binding to the promoter and thereby stimulates transcription (Kolb et al., 1993; Busby and Ebright, 1994; Benoff et al., 2002; Lawson et al., 2004). One of the best characterized Class I promoters is the *P*_*lac*_ promoter, which has a cAMP-CRP binding site centered at position −61.5 relative to the transcriptional start site defined as +1. In Class II promoters, the cAMP-CRP binding site overlaps the core promoter −35 element (Ushida and Aiba, 1990). At these promoters there are three surfaces on the DNA-bound cAMP-CRP complex that interact with RNAP. Besides the AR1-αCTD interaction that facilitates RNAP binding, activation region 2 (AR2) interacts with the N-terminal domain of the RNAP α subunit (αNTD), and the activation region 3 (AR3) interacts with region 4 of the σ^70^ subunit (Lawson et al., 2004). These two additional interactions facilitate transcription initiation at a post-binding step (Niu et al., 1996; Lawson et al., 2004). One of the best characterized Class II CRP-dependent promoters is the *P1*_*gal*_ promoter which has a cAMP-CRP binding site centered at position −41.5 (Kolb et al., 1993).

In addition to regulating sugar metabolism, cAMP-CRP regulates natural competence in *Haemophilus influenzae* and *Vibrio cholerae* together with the transcriptional co-regulator Sxy (Redfield et al., 2005; Cameron and Redfield, 2006; Lo Scrudato and Blokesch, 2012). Natural competence is the ability of a bacterium to take up exogenous DNA and incorporate it into its own genome. While this is a common feature of many Gram-positive and Gram-negative bacteria, natural competence has not been observed in *E. coli*.

In *H. influenzae* the genes necessary for competence are preceded by a DNA motif termed a CRP-S site. CRP-S sites are similar to canonical palindromic CRP binding sites, but typically differ at the middle position within each half site 5′-T_4_G_5_**C**_6_G_7_A_8_-6N-T_15_C_16_**G**_17_C_18_A_19_-3′ (Cameron and Redfield, 2006). Although this nucleotide position does not make a specific contact with cAMP-CRP, the T:A → C:G mutation is known to reduce cAMP-CRP binding ∼80-fold (Chen et al., 2001). Activation of promoters by cAMP-CRP bound to CRP-S sites requires the presence of Sxy; however, the molecular mechanism behind this requirement has remained unknown (Cameron and Redfield, 2006).

*Escherichia coli* has homologs to all but one of the genes necessary for competence development in *H. influenzae*, including Sxy (Cameron and Redfield, 2006; Sinha et al., 2009). It has also been shown that most of the competence genes found in *E. coli* are preceded by DNA motifs homologous to the CRP-S sites found in *H. influenzae*, and that induction of these are dependent on cAMP-CRP and Sxy as well (Sinha et al., 2009). The promoter of *sxy* itself has been shown to contain a CRP-S site in *E. coli* and is thus positively autoregulated at the level of transcription (Jaskolska and Gerdes, 2015).

With the purpose of understanding the molecular interactions taking place between cAMP-CRP, Sxy, and the RNAP at the CRP-S sites inducing transcriptional activation, we performed a series of genetic screens. We found that residues Q194 and L196 of CRP are specifically required for the activation of CRP-S promoters but are dispensable for other CRP functions. Amino acid changes in AR1, but not in AR2, of CRP also eliminated the activation of CRP-S promoters. The mutation CRP Q194R could be specifically suppressed by an amino acid change in Sxy (S30C), suggesting a direct interaction between the two proteins. Analyses of the CRP binding sites at CRP-S promoters suggest that these sites carry specific information for Sxy-mediated activation. Moreover, we identified constitutive Sxy mutants, causing higher levels of promoter activation than WT Sxy. Based on our results we suggest a model where cAMP-CRP and Sxy interact directly, while cAMP-CRP contacts RNAP in a Class I like manner.

## Results

### Genetic Screen for CRP Mutants Defective in Activation of “CRP-S” Promoters

**FIGURE 1:**
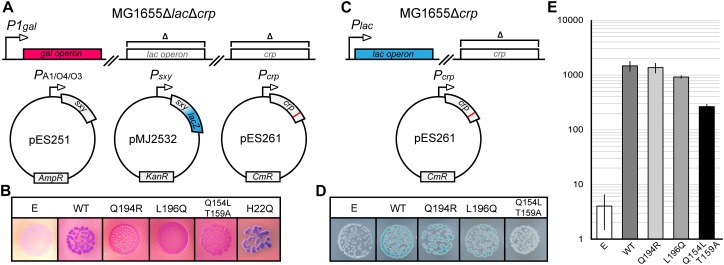
Isolation of CRP mutants defective in activation of CRP-S promoters. **(A)** Schematic overview of the experimental setup for isolation of CRP mutants with a reduced ability to induce CRP-S sites. The *crp* coding sequence was mutated by error-prone PCR in plasmid pES261 and mutants were screened in MG1655 Δ*lac*Δ*crp* cells (Jaskolska and Gerdes, 2015) carrying plasmids pMJ2532 (Jaskolska and Gerdes, 2015) and pES251 (for Sxy production). Galactose fermentation (red color) was used as a control for CRP function (activation of Class II promoters) and the LacZ reporter in pMJ2532 (blue color) was used to assess activation of Sxy dependent promoters. **(B)** Phenotypes of CRP mutants on MacConkey galactose X-gal plates. Cells carrying an empty vector (E, p15A) are white because none of the reporters are activated in the absence of CRP, while cells carrying wild-type (WT) *crp* are purple as both promoters are active. Cells with CRP mutants Q194R, L196Q, and Q154L T159A appear red, indicating galactose fermentation but no LacZ production. CRP H22Q has the opposite phenotype. **(C)** Schematic overview of the setup used to assay the activation of the Class-I *P*_*lacZ*_ promoter. The native *lac* operon of MG1655Δ*crp* is used as a reporter in the presence of plasmids borne CRP mutants. **(D)** Activation of the chromosomal Class I (*P*_*lacZ*_) promoter by CRP mutants defective in the activation of CRP-S promoters. The plasmid borne AR1 CRP mutant Q154L T159A is not able to activate the *lacZ* promoter, and cells are white on X-gal plates. Cells containing WT CRP, CRP(Q194R), or CRP(L196Q) appear blue. **(E)** Quantification of chromosomal *lac*Z expression in the presence of CRP mutants Q194R and L196Q. Cells were grown in M9 medium containing glycerol (0.8%) and Casamino acids (0.2%) in a shaking incubator at 37°C. β-Galactosidase assays were performed as described previously (Miller, 1972). Results shown are the averages of measurements of three biological replicates. Error bars indicate ±1 SEM.

Previous studies hypothesized that Sxy binds cAMP-CRP directly to facilitate its binding to the otherwise low affinity CRP-S sites (Redfield et al., 2005; Cameron and Redfield, 2006; Sinha et al., 2009). We therefore reasoned that, if such a direct Sxy-CRP interaction exists, it might be possible to identify CRP mutants which are specifically defective in the activation of CRP-S promoters due to an abrogation of Sxy binding. In order to test this hypothesis, we performed a random mutagenesis of the *crp* coding sequence and probed the resulting mutant library in the double reporter system outlined in Figure 1A. As a reporter for activation of CRP-S promoters we used a plasmid-borne translational *P*_*sxy*_:*lacZ* fusion (Jaskolska and Gerdes, 2015), while the general functionality of *crp* mutants was evaluated based on the activity of the chromosomal *gal* operon. *sxy* was expressed from the *lac*-derived isopropyl β-D-1-thiogalactopyranoside (IPTG) inducible promoter *P_*A*__1__/O__4__/__03_*, previously shown to yield high levels of Sxy without the toxic effects otherwise associated with *sxy* overexpression (Jaskolska and Gerdes, 2015). Overexpression of *sxy* causes a strong induction of the *P*_*sxy*_:*lacZ* fusion, thus making it easier to detect potential changes in CRP-S induction upon visual inspection (Jaskolska and Gerdes, 2015). Cells were grown on MacConkey agar plates containing galactose and 5-bromo-4-chloro-3-indolyl-β-D-galactopyranoside (X-gal). Cells carrying a fully functional *crp* allele appear purple on these plates (Figure 1B, WT) due to the expression of (i) LacZ that converts X-gal to a blue substance, and (ii) the *gal* operon that leads to red coloring on the MacConkey plates due to fermentation of galactose. As we were interested in mutants that are defective only in the activation of CRP-S promoters, we screened for red colonies which expressed the *gal* operon but did not produce LacZ. Seventeen red colonies were isolated and sequenced.

Analysis of the DNA sequences of *crp* mutants isolated from red colonies identified four amino acid substitutions that interfered with activation of CRP-S promoters (Q154L, T159A, Q194R, and L196Q, Figure 1B). Two of these falls within the AR1 of CRP (Q154L and T159A) (Zhou et al., 1993) and were found to be deficient in activation of the Class I *lac* promoter as well (Figures 1C–E). These positions are thus not considered to be specifically essential for CRP-S dependent induction. However, mutations Q194R and L196Q fall into a region which has not so far been characterized and appear to activate the Class I *lac* promoter similar to WT CRP (Figures 1D,E). Further site-directed mutagenesis of these positions showed that substitutions Q194E/H/K/L and L196I/N/P/R/S/T all eliminate the ability of CRP to activate CRP-S promoters while substitution Q194W behaves as WT (Supplementary Figures S1, S2).

Interestingly a few blue colonies also appeared on the screening plates signifying either the loss of Class II *P1*_*gal*_ induction but retention of CRP-S induction or increased CRP-S induction (Figure 1). Sequencing one of these *crp* alleles identified amino acid substitution H22Q (Figure 1B), which is located in the AR2 of CRP (Niu et al., 1996). Mutating this residue has been shown to reduce activation of Class II promoters without affecting Class I promoters (Niu et al., 1996). Based on these findings we propose that activation of CRP-S promoters requires the AR1 of CRP and that the AR2 does not seem to be critical for this process.

### A Genetic Screen for Suppressor Sxy Mutants for CRPQ194R

**FIGURE 2:**
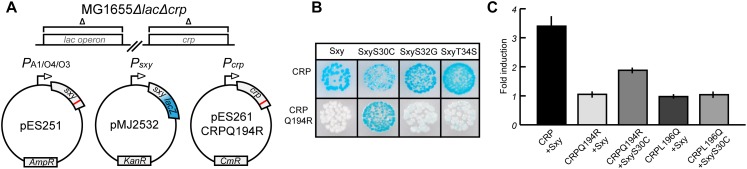
Isolation of Sxy mutants that suppress the inability of CRP(Q194R) to induce CRP-S promoters. **(A)** Schematic overview of the experimental setup. The *sxy* coding sequence was mutated by error-prone PCR and cloned into pMG25 creating pES251 and mutants were screened in MG1655 Δ*lac*Δ*crp* cells (Jaskolska and Gerdes, 2015) carrying plasmids pMJ2532 (Jaskolska and Gerdes, 2015) and pES261CRPQ194R (for CRP Q194R production). The LacZ reporter in pMJ2532 (blue color) was used to assess activation of Sxy dependent promoters. **(B)** Phenotypes of suppressor Sxy mutants. Sxy S30C shows strong activation of the reporter while S32G and T34S have slightly higher activity than wild-type (WT) Sxy. **(C)** Quantification of suppression by Sxy S30C using a *P_*sxy*_:yfp* reporter. Sxy S30C activates the CRP-S promoter in the presence of CRP Q194R but not of CRP L196Q. The quantifications ± 1 SD are the result of three biological replicates as described in the section “Materials and Methods.”

Assuming that amino acids Q194 and L196 of CRP mark the interface that interacts with Sxy, we attempted to isolate *sxy* mutants that restored activation of CRP-S promoters in cells expressing the CRP(Q194R) protein. We used a similar screening setup as in previous experiments (Figure 1), except that this time the *sxy* coding sequence was mutated (Figure 2A). Three single amino acid substitutions were identified in Sxy that showed higher activity of the CRP-S promoter reporter than WT Sxy (Figure 2B; bottom panel). The strongest activation was obtained with Sxy(S30C). All three mutants were able to activate CRP-S promoters in the presence of WT CRP, although Sxy(S30C) appeared heterogeneous on plate (Figure 2B; top panel). Quantification of CRP-S promoter activities using a translational *P*_*sxy*_:*yfp* fusion showed Sxy(S30C)-mediated transcriptional activation in combination with CRP(Q194R) but not with CRP(L196Q) (Figure 2C). These results indicate that Sxy(S30C) is a specific suppressor of CRP(Q194R), suggesting a direct interaction of CRP and Sxy (Rhodius and Busby, 2000).

### Isolation of Constitutive Sxy Mutants

**FIGURE 3:**
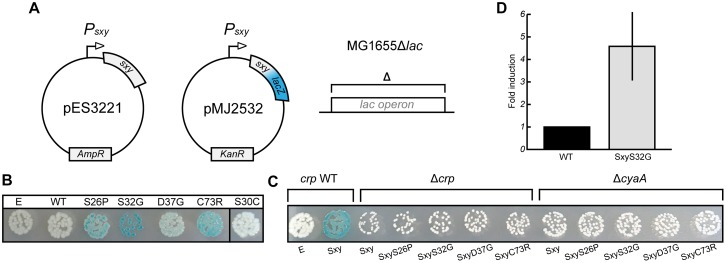
Isolation of constitutive Sxy mutants. **(A)** Schematic overview of the experimental setup to find Sxy mutants that is more active than wild-type (WT). The *sxy* coding sequence was mutated by error-prone PCR in plasmid pES3221 and the mutant library was transferred into MG1655Δ*lac* cells carrying a plasmid borne *P*_*sxy*_-*lacZ* reporter (pMJ2532) (Jaskolska and Gerdes, 2015). **(B)** Phenotypes of four constitutive mutants, S26P, S32G, D37G, and C73R on LB X-gal plates. Cells carrying an empty vector (E, pES3221 without *sxy*) and pES3221 with WT *sxy* are shown for reference. SxyS30C, isolated as a suppressor for CRPQ194R, is not constitutive. **(C)** Activation of “CRP-S” promoters by constitutive *sxy* mutants depends on CRP and cAMP, as no LacZ activity was observed in MG1655Δ*lac*Δ*crp*/pMJ2532 and MG1655Δ*lac*Δ*cyaA*/pMJ2532 cells. **(D)** Activity of the chromosomal *sxy*S32G allele relative to WT. Sxy activity was quantified in MG1655Δ*lac* and MG1655Δ*lac sxy*S32G cells using a plasmid borne *P_*sxy*_-yfp* reporter (pES91). The quantifications ± 1 *SD* are the results of three biological replicates as described in the section “Materials and Methods.”

*E. coli* Sxy is expressed at a very low level in log phase cells in rich medium (LB broth), but the transcriptional auto-activation of *sxy* can be exploited to induce expression of the chromosomal *sxy* gene upon ectopic expression of Sxy in the presence of cAMP-CRP (Jaskolska and Gerdes, 2015). However, the physiological conditions that lead to auto-activation of the chromosomal *sxy* gene have not yet been identified. We assumed that Sxy activity is regulated allosterically by an unknown signal. If this were true, it should be possible to identify Sxy mutants that fold similar to the allosterically activated conformation and are locked “on” (Harman et al., 1986; Suckow et al., 1996). We thus performed a random mutagenesis of the *sxy* coding sequence under the control of its native promoter and screened for mutants that showed stronger activation of the plasmid-borne translational *P_*sxy*_:lacZ* fusion (Figure 3A). Four single amino acid substitutions were found that made Sxy more active (S26P, S32G, D37G, and C73R; Figure 3B). All of the four mutants functioned in a cAMP- and CRP-dependent manner, since no activation of the CRP-S promoter by these mutants was detected in Δ*cyaA* or Δ*crp* cells (Figure 3C). As Sxy(S32G) showed the strongest auto-activation, the point mutation responsible for this substitution was introduced into the chromosomal *sxy* gene. The chromosomal *sxy* mutant showed ∼4.5-fold higher activation of the plasmid-borne translational *P_*sxy*_:yfp* fusion compared to WT *sxy* (Figure 3D).

### Functionality of the N-Terminal Domain of Sxy

**FIGURE 4:**
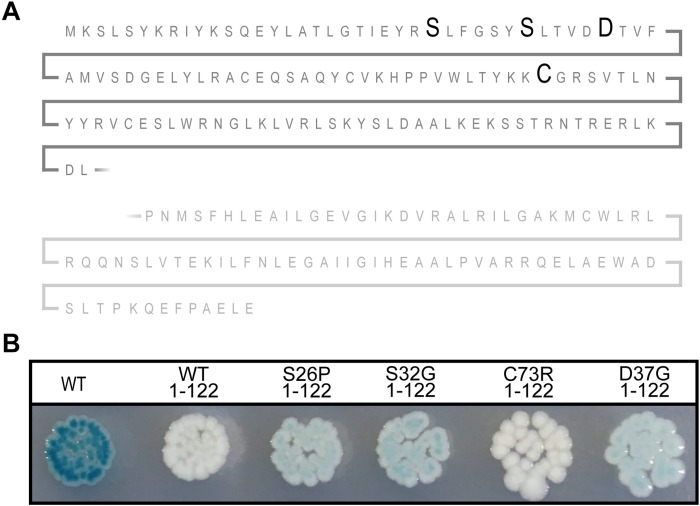
The N-terminal of constitutive Sxy mutants remains active. **(A)** Schematic of the Sxy N-terminal. The position of mutations S26P, S32G, D37G, and C73R are marked with bold letters. The place of truncation is marked with a broken line. **(B)** Activities of the truncated Sxy proteins were assessed using a P*_*sxy*_*:*lacZ* fusion. MG1655Δ*lac* pMJ2532 (Jaskolska and Gerdes, 2015) cells expressing wild-type Sxy (WT, pES251) or its truncated derivatives, Sxy_1__–__122_ (pES251Sxy_1__–__122_), SxyS26P_1__–__122_ (pES251SxyS26P_1__–__122_), SxyS32G_1__–__122_ (pES251SxyS32G_1__–__122_), SxyC73R_1__–__122_ (pES251SxyC73R_1__–__122_), or SxyD37G_1__–__122_ (pES251SxyD37G_1__–__122_), were grown on LB agar plates containing X-gal, IPTG, and appropriate antibiotics.

All the mutations in *sxy* that affected the regulation of CRP-S promoters caused amino acid substitutions close to the N-terminus of Sxy. Because the molecular structure of full-length Sxy has not been elucidated yet, we used the Phyre2 software to create a structure prediction (Kelley et al., 2015). In this prediction, Sxy had separate N- and C-terminal domains that were connected by a flexible linker. We were therefore curious whether the predicted N-terminal domain would be able to activate CRP-S promoters in the absence of the C-terminal domain. Consequently, we created a plasmid expressing the predicted N-terminal domain of Sxy together with the flexible region (Sxy_1__–__122_). Although WT Sxy_1__–__122_ appears inactive (Figure 4), introduction of the substitutions resulting in constitutive sxy expression (S26P, S32G, or D37G) into the truncated protein partially restored its functionality. However, Sxy_1__–__122_C73R remained inactive, suggesting that this residue acts by a different mechanism than the other three.

### Analysis of the CRP-S Site

**FIGURE 5:**
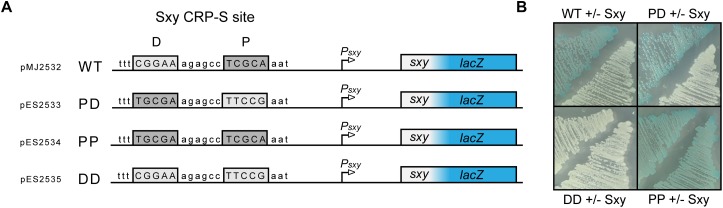
Both half sites of the *E. coli* CRP-S site are required for Sxy dependent induction, but the orientation of the site has no effect. **(A)** Schematic drawing of the *P*_*sxy*_-*lacZ* reporter region in plasmid pMJ2532 (Jaskolska and Gerdes, 2015), and of its variants differing in the CRP-S half sites. The promoter proximal half-site is marked P and the promoter distal half-site is marked D. **(B)** Activity of the *P*_*sxy*_-*lacZ* reporter in the four different constructs in MG1655Δ*lac* cells with or without Sxy expression from plasmid pES251.

The CRP-S binding motif in *E. coli* described by Sinha et al. (2009) comprises two distinct half sites (5′-T_4_G_5_**C**_6_G_7_A_8_-6N-T_15_T_16_C_17_C_18_A_19_-3′) of which one resembles a canonical CRP half site with a T_6_→C_6_ in the middle position, while the other half site is less conserved. This means that, unlike CRP-S sites in *H. influenzae*, the CRP-S sites of *E. coli* are not palindromic (Sinha et al., 2009). However, upon closer inspection of the various CRP-S sites discovered by Sinha et al. (2009) there is no apparent preference in regard to the orientation of the two half sites relative to the promoter. The CRP-S site of the *sxy* promoter is oriented with the conserved half site in the promoter proximal position and the other in the promoter distal position, as shown in Figure 5A. In order to determine if the orientation of the two half sites relative to the promoter and the surrounding sequence had any effect on Sxy-dependent CRP-S induction, three additional *P*_*sxy*_:*lacZ* reporters were constructed (Figure 5A). The CRP-S site was reverse complemented (PD), substituted by two of the conserved (5′-TGCGA-3′) half-sites (PP) or by two of the non-conserved (5′-TTCCG-3′) half-sites (DD). MG1655Δ*lac* cells carrying the different reporter constructs and plasmid pES251 were grown on indicator plates in the presence and absence of Sxy expression (Figure 5B). In the presence of a CRP-S site, Sxy induced reporter expression regardless of the orientation of the site relative to the promoter (WT and PD). However, the construct carrying two non-conserved half-sites (DD) did not show any reporter activity upon Sxy expression. Contrarily, having two of the conserved half-sites (PP) led to a high reporter activity, regardless of the level of Sxy expression. These findings suggest (i) that Sxy-dependent promoter activation relies on a sequence or structural information within the CRP-S site, (ii) that the presence of the two different half sites is required, and (iii) that the orientation of the half sites relative to the transcriptional start site is not relevant.

## Discussion

### Protein–Protein Interactions in the Regulation of “CRP-S” Promoters

The aim of this study was to explore and characterize the molecular interactions taking place at the CRP-S-dependent promoters of *E. coli* using a genetic approach. We found mutations within the AR1 of CRP abolishing induction of the CRP-S promoter as well as the Class I *Plac* promoter, and mutations within the AR2 reducing induction of the Class II *P1*_*gal*_ promoter without affecting the CRP-S promoter. This supports the hypothesis that CRP-S promoters are activated by a mechanism similar to what is observed at Class I promoters. This interpretation is also in accordance with the observation that the distance between CRP-S sites and the transcriptional start site is similar to the distance typical for Class I promoters (Cameron and Redfield, 2006; Sinha et al., 2009). Furthermore, we identified a novel surface on the CRP protein, here named Sxy Interacting Region (SIR), which is specifically required for activation of CRP-S promoters but is dispensable for the activation of regular Class I and Class II promoters. This surface was defined by amino acid substitutions at residues Q194 and L196. The lost activation of CRP-S promoters in the CRP(Q194R) mutant could be restored by a specific amino acid substitution in Sxy(S30C). The observation that Sxy(S30C) is a specific suppressor of CRP(Q194R) (Figure 2B) suggests that the novel surface identified on the CRP protein is engaging in a direct and specific interaction with Sxy. Additionally, we found *sxy* mutants that showed increased CRP-S promoter activation in the presence of WT *crp*. The strongest constitutive substitution found was S32G (Figure 3). Interestingly, both Q194 and L196 in CRP, as well as G32 in Sxy (TfoX) are conserved in *Haemophilus* and *Vibrio* species, which are known to be naturally competent (Supplementary Figure S3). The proximity of the two sites in Sxy, one defining the interaction surface with CRP(S30C), and the other causing Sxy hyperactivity (S32G), may indicate a competitive regulatory mechanism for Sxy activity, where a so far unknown molecule would compete with the SIR site of CRP for the S26-D37 region of Sxy. This hypothesis is further supported by the observation that the C-terminal truncation of Sxy(S32G) is functional, while the same truncation in WT Sxy is inactive. However, it is worth noting that CRP regulates the transcription of as many as 70 transcription factor (TF) genes (Shimada et al., 2011). Although no positive regulators for sxy transcription other than Sxy has been identified, we cannot exclude contribution of indirect effects through CRP/Sxy-mediated regulation of TFs or small molecule levels that would influence the activity of TFs.

### Does Sxy Bind DNA?

**FIGURE 6:**
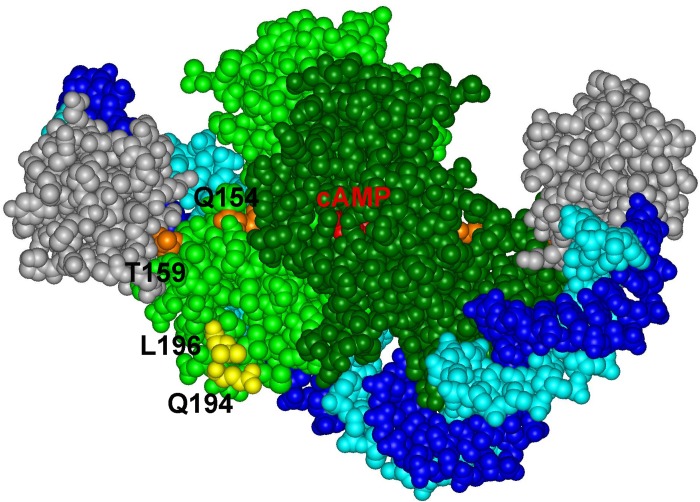
Positions of CRP residues Q194 and L196 on the complex of the CRP dimer (light and dark green) with DNA (light and dark blue) and the alpha subunit of RNA polymerase (gray) (PDB: 5CIZ).

The Sxy protein does not contain any recognizable DNA binding domain and no sequence conservation outside the cAMP-CRP binding sites at CRP-S promoters in *E. coli* has been described (Cameron and Redfield, 2006; Sinha et al., 2009). Therefore, if Sxy makes specific contacts with DNA, these contacts should occur within the DNA sequence recognized by cAMP-CRP. The CRP-S sites indeed carry characteristic sequence features that seem to be required for Sxy-mediated activation. The CRP-S sites show an asymmetric organization, carrying one half site with a higher affinity for CRP binding than the other. This feature seems to be crucial for their function since replacing the higher affinity half site with the lower affinity one or vice versa eliminates Sxy-mediated promoter activation. However, the orientation of the two sites is not important. Based on these observations we suggest that recognition of CRP-S sites by the cAMP-CRP-Sxy complex involves specific DNA–protein interactions that do not occur at the CRP-S sites in the absence of Sxy or at regular CRP-binding sites. It has previously been hypothesized that the asymmetry of the CRP-S motif may affect CRP’s ability to bind as a dimer and thus create a need for Sxy to facilitate CRP-DNA binding to one half site (Sinha et al., 2009). The CRP residues Q194 and L196, which were found to potentially interact with Sxy in our genetic screens, are located on the surface of CRP, close to the bound DNA (Figure 6). In principle, this topology would allow Sxy to contact CRP and the CRP bound DNA at the same time, which could explain the requirement for the specific, weak half site at Sxy regulated promoters.

## Conclusion

Our study supports a model involving (i) Class I-type activation of transcription by cAMP-CRP, (ii) direct interaction cAMP-CRP and Sxy, and (iii) possibly a contact between Sxy and DNA within the 16-bp cAMP-CRP-binding site.

## Materials and Methods

### Strains, Plasmids, and Growth Conditions

Strain and plasmid construction was done by standard laboratory methods as described in the section “Materials and Methods” in the Supplementary Material. Strains, plasmids, and oligonucleotides used in this study are listed in Supplementary Tables S1–S3, respectively. DNA sequencing was performed by Eurofins Genomics. Cells were routinely grown at 37°C in LB or MOPS 0.4% D-ribose media shaking, or on LB agar plates. The following antibiotics were used when appropriate: ampicillin 100 μg ml^–1^, chloramphenicol 50 μg ml^–1^, kanamycin 25 μg ml^–1^, and tetracycline 20 μg ml^–1^. Expression from LacI-regulated promoters was induced by the addition of 1 mM of IPTG. To visualize β-galactosidase activity, plates were supplemented with X-gal at a final concentration of 40 μg ml^–1^.

### Quantification of Gene Expression

Exponential phase cultures were diluted in MOPS 0.4% ribose medium in 96-well plates. Wells for induction of *sxy* expression also contained 1 mM IPTG. Cells were then grown at 37°C for 24 h shaking in a temperature-controlled plate reader (Synergy H1, BioTek). OD_600_ and YFP fluorescence intensity (ex 479 nm/em 520 nm) were measured every 15 min. Fluorescence values were normalized to OD_600_ and corrected with the auto-fluorescence of MG1655 cells. Relative expression of *sxy* in MG1655Δ*lac* pES91 and MG1655Δ*lac*SxyS32G pES91 was estimated by growing cells in culture tubes until OD_600_ 0.8–1 then diluting 1/10 into 96-well plates and measuring YFP fluorescence using a plate reader. YFP values were normalized to OD_600_ and corrected with the auto-fluorescence of MG1655Δ*lac* cells.

### Genetic Screen for CRP Mutants

A DNA fragment containing the *crp* open-reading frame was amplified and mutated using Error-prone PCR (Rasila et al., 2009) using primers ORFCRPCW and CRP*Xho*ICCW. The mutated DNA was cloned into pES261 using restriction sites *Hin*dIII and *Xho*I creating a library of mutated *crp* under control of the natural *crp* promoter. The mutant library was electroporated into MG1655Δ*lac*Δ*crp* cells already carrying plasmids pMJ2532 (Jaskolska and Gerdes, 2015) and pES251. Transformed cells were selected and screened on MacConkey galactose agar containing X-gal, IPTG, chloramphenicol, kanamycin, and ampicillin. The pES261 plasmid variants were isolated from the selected colonies and the DNA sequence of the *crp* open-reading frame was determined. All point mutations found were individually transferred to a clean pES261 background by site-directed mutagenesis (Liu and Naismith, 2008) and transferred into MG1655Δ*lac*Δ*crp* cells carrying the pMJ2532 and pES251 plasmids to verify which mutations are responsible for the observed colony phenotypes.

### Genetic Screen for Suppressor Sxy Mutants

A DNA fragment containing *sxy* was amplified and mutated by Error-prone PCR (Rasila et al., 2009) using primers sxycw*Bam*HI and sxyccw*hin*dIII. To create a mutant *sxy* library, the amplified and mutated DNA fragments were digested by *Bam*HI and *Hin*dIII and inserted between the same sites in plasmid pMG25 creating pES251. The mutant library was electroporated into MG1655Δ*lac*Δ*crp* cells carrying plasmids pMJ2532 (Jaskolska and Gerdes, 2015) and pES261CRPQ194R. Transformed cells were selected and screened on LB agar containing X-gal, IPTG, chloramphenicol, kanamycin, and ampicillin. The pES251 plasmid variants were isolated from the selected colonies and the DNA sequence of the *sxy* open-reading frame was determined. All point mutations found were individually transferred to a clean pES251 by site-directed mutagenesis (Liu and Naismith, 2008) and transferred into MG1655Δ*lac*Δ*crp* cells carrying the pMJ2532 and pES261CRPQ194R plasmids to verify which mutations are responsible for the displayed colony phenotypes.

### Screen for Constitutive Sxy Mutants

A DNA fragment containing the sxy open-reading frame was amplified and mutated by Error-prone PCR (Rasila et al., 2009) using primers ORFsxyCW and sxyccwhindIII. The mutated DNA was cloned into pES3221 using restriction sites *Hin*dIII and *Bam*HI, creating a library of mutated sxy transcribed by the natural sxy promoter. The mutant library was transferred into MG1655Δlac pMJ2532 (Jaskolska and Gerdes, 2015) cells by electroporation. Transformed cells were selected and screened on LB agar containing X-gal, ampicillin, and kanamycin. Plasmids were isolated from colonies displaying a blue phenotype and mutations in the sxy open-reading frame were identified by DNA sequencing. Point mutations found were transferred individually to clean pES3221 using site-directed mutagenesis PCR (Liu and Naismith, 2008) and MG1655Δlac pMJ2532 was transformed with the mutated plasmids to identify mutations responsible for the displayed phenotype.

## Data Availability

All data used and created in this study, not presented in the manuscript, can be found in the Supplementary Material.

## Author Contributions

ES, KG, and SS conceived the idea. ES and SS designed the experimental setup and wrote the manuscript. ES primarily carried out the acquisition of data. AS performed the β-galactosidase assays. ES, AS, and SS performed the analysis and interpretation of the data.

## Conflict of Interest Statement

The authors declare that the research was conducted in the absence of any commercial or financial relationships that could be construed as a potential conflict of interest.

## References

[B1] Aiba H. Adhya S. de Crombrugghe B. (1981). Evidence for two functional gal promoters in intact *Escherichia coli* cells. *J. Biol. Chem.* 256 11905–11910. 6271763

[B2] Benoff B. Yang H. Lawson C. L. Parkinson G. Liu J. Blatter E. (2002). Structural basis of transcription activation: the CAP-alpha CTD-DNA complex. *Science* 297 1562–1566. 10.1126/science.1076376 12202833

[B3] Busby S. Ebright R. H. (1994). Promoter structure, promoter recognition, and transcription activation in prokaryotes. *Cell* 79 743–746. 10.1016/0092-8674(94)90063-9 8001112

[B4] Busby S. Ebright R. H. (1999). Transcription activation by catabolite activator protein (CAP). *J. Mol. Biol.* 293 199–213. 10.1006/jmbi.1999.3161 10550204

[B5] Cameron A. D. Redfield R. J. (2006). Non-canonical CRP sites control competence regulons in *Escherichia coli* and many other gamma-*proteobacteria*. *Nucleic Acids Res.* 34 6001–6014. 10.1093/nar/gkl734 17068078 PMC1635313

[B6] Chen S. F. Gunasekera A. Zhang X. P. Kunkel T. A. Ebright R. H. Berman H. M. (2001). Indirect readout of DNA sequence at the primary-kink site in the CAP-DNA complex: alteration of DNA binding specificity through alteration of DNA kinking. *J. Mol. Biol.* 314 75–82. 10.1006/jmbi.2001.5090 11724533

[B7] Grainger D. C. Hurd D. Harrison M. Holdstock J. Busby S. J. (2005). Studies of the distribution of *Escherichia coli* cAMP-receptor protein and RNA polymerase along the *E. coli chromosome*. *Proc. Natl. Acad. Sci. U.S.A.* 102 17693–17698. 10.1073/pnas.0506687102 16301522 PMC1308901

[B8] Harman J. G. McKenney K. Peterkofsky A. (1986). Structure-function analysis of three cAMP-independent forms of the cAMP receptor protein. *J. Biol. Chem.* 261 16332–16339. 3023348

[B9] Hunziker A. Tuboly C. Horvath P. Krishna S. Semsey S. (2010). Genetic flexibility of regulatory networks. *Proc. Natl. Acad. Sci. U.S.A.* 107 12998–13003. 10.1073/pnas.0915003107 20615961 PMC2919941

[B10] Jaskolska M. Gerdes K. (2015). CRP-dependent positive autoregulation and proteolytic degradation regulate competence activator Sxy of *Escherichia coli*. *Mol. Microbiol.* 95 833–845. 10.1111/mmi.12901 25491382

[B11] Kelley L. A. Mezulis S. Yates C. M. Wass M. N. Sternberg M. J. E. (2015). The Phyre2 web portal for protein modeling, prediction and analysis. *Nat. Protoc.* 10:845. 10.1038/nprot.2015.053 25950237 PMC5298202

[B12] Kolb A. Busby S. Buc H. Garges S. Adhya S. (1993). Transcriptional regulation by cAMP and its receptor protein. *Annu. Rev. Biochem.* 62 749–795. 10.1146/annurev.biochem.62.1.749 8394684

[B13] Lawson C. L. Swigon D. Murakami K. S. Darst S. A. Berman H. M. Ebright R. H. (2004). Catabolite activator protein: DNA binding and transcription activation. *Curr. Opin. Struct. Biol.* 14 10–20. 10.1016/j.sbi.2004.01.012 15102444 PMC2765107

[B14] Lee D. J. Busby S. J. (2012). Repression by cyclic AMP receptor protein at a distance. *MBio* 3 e289–e212. 10.1128/mBio.00289-12 22967981 PMC3445967

[B15] Liu H. Naismith J. H. (2008). An efficient one-step site-directed deletion, insertion, single and multiple-site plasmid mutagenesis protocol. *BMC Biotechnol.* 8:91. 10.1186/1472-6750-8-91 19055817 PMC2629768

[B16] Lo Scrudato M. Blokesch M. (2012). The regulatory network of natural competence and transformation of *Vibrio cholerae*. *PLoS Genet.* 8:e1002778. 10.1371/journal.pgen.1002778 22737089 PMC3380833

[B17] Miller J. H. (1972). *Experiments in Molecular Genetics, Cold Spring Harbor Laboratory.* New York, NY: Cold Spring Harbor.

[B18] Niu W. Kim Y. Tau G. Heyduk T. Ebright R. H. (1996). Transcription activation at class II CAP-dependent promoters: two interactions between CAP and RNA polymerase. *Cell* 87 1123–1134. 10.1016/s0092-8674(00)81806-1 8978616 PMC4430116

[B19] Rasila T. S. Pajunen M. I. Savilahti H. (2009). Critical evaluation of random mutagenesis by error-prone polymerase chain reaction protocols, *Escherichia coli* mutator strain, and hydroxylamine treatment. *Anal. Biochem.* 388 71–80. 10.1016/j.ab.2009.02.008 19454214

[B20] Redfield R. J. Cameron A. D. Qian Q. Hinds J. Ali T. R. Kroll J. S. (2005). A novel CRP-dependent regulon controls expression of competence genes in *Haemophilus influenzae*. *J. Mol. Biol.* 347 735–747. 10.1016/j.jmb.2005.01.012 15769466

[B21] Rhodius V. A. Busby S. J. (2000). Interactions between activating region 3 of the *Escherichia coli* cyclic AMP receptor protein and region 4 of the RNA polymerase sigma(70) subunit: application of suppression genetics. *J. Mol. Biol.* 299 311–324. 10.1006/jmbi.2000.3737 10860740

[B22] Shimada T. Fujita N. Yamamoto K. Ishihama A. (2011). Novel roles of cAMP receptor protein (CRP) in regulation of transport and metabolism of carbon sources. *PLoS One* 6:e20081. 10.1371/journal.pone.0020081 21673794 PMC3105977

[B23] Sinha S. Cameron A. D. Redfield R. J. (2009). Sxy induces a CRP-S regulon in *Escherichia coli*. *J. Bacteriol.* 191 5180–5195. 10.1128/JB.00476-09 19502395 PMC2725579

[B24] Suckow J. Markiewicz P. Kleina L. G. Miller J. Kisters-Woike B. Muller-Hill B. (1996). Genetic studies of the Lac repressor. *XV:* 4000 single amino acid substitutions and analysis of the resulting phenotypes on the basis of the protein structure. *J. Mol. Biol.* 261 509–523. 10.1006/jmbi.1996.0479 8794873

[B25] Ushida C. Aiba H. (1990). Helical phase dependent action of CRP: effect of the distance between the CRP site and the -35 region on promoter activity. *Nucleic Acids Res.* 18 6325–6330. 10.1093/nar/18.21.6325 2173826 PMC332499

[B26] Zheng D. Constantinidou C. Hobman J. L. Minchin S. D. (2004). Identification of the CRP regulon using in vitro and in vivo transcriptional profiling. *Nucleic Acids Res.* 32 5874–5893. 10.1093/nar/gkh908 15520470 PMC528793

[B27] Zhou Y. H. Zhang X. P. Ebright R. H. (1993). “Identification of the activating region of catabolite gene activator protein (Cap) - isolation and characterization of mutants of cap specifically defective in transcription activation. *Proc. Natl. Acad. Sci. U.S.A.* 90 6081–6085. 10.1073/pnas.90.13.6081 8392187 PMC46871

