# Supplementary material for: CRP Interacts Specifically With Sxy to Activate Transcription in Escherichia coli
Source: Front Microbiol. 2019 Aug 30;10:2053. doi: 10.3389/fmicb.2019.02053 (PMC6728893; doi:10.3389/fmicb.2019.02053)
Supplement: Supplementary file 1 [file Data_Sheet_1.docx]

Supplementary Material

# Supplementary Materials and Methods

## Strain construction

Strain ES10 was created by introducing a single amino acid change into Sxy using scar-less recombineering as described by reference (Blank et al., 2011). Primers sxypwrg100cw and sxypwrg100ccw was used to amplify the insertion cassette, primers sxymutrekfragcw and sxymutrekfragccw was used to amplify DNA containing the SxyS32G mutation from pES251SxyS32G and primers 500upsxycw and sxyccw*hin*dIII was used to check for correct insertion and removal of cassette/mutation

## Plasmid construction

**Plasmid pES251** was created by amplifying the open reading frame of *sxy* from *E. coli* K-12 MG1655 using primers sxycw*Bam*HI + sxyccw*hin*dIII, then digesting fragment as well as vector (pMG25) with restriction enzymes *Bam*HI and *Hin*dIII. The vector was treated with alkaline phosphatase to inhibit re-ligation. The insert was then ligated into the vector using T4 ligase.

**Plasmid pES3221** was created by amplifying *sxy* and 500 bp of its upstream sequence from *E. coli* K-12 MG1655 using primers 500upsxy + sxyccw*hin*dIII, then digesting fragment as well as vector (pBR322) with restriction enzymes *Eco*RI and *Hin*dIII. The vector was treated with alkaline phosphatase to inhibit re-ligation. The insert was then ligated into the vector using T4 ligase.

**Plasmid pES261** was created by amplifying *crp* and 500 bp of its upstream sequence from *E. coli* K-12 MG1655 using primers 500upCRPCW + CRP*Xho*ICCW, then digesting fragment as well as vector p15A with restriction enzymes *Bgl*II and *Xho*I. The vector was treated with alkaline phosphatase to inhibit re-ligation. The insert was then ligated into the vector using T4 ligase, replacing the dCas9 gene with *crp*.

**Plasmid pES2533** was created by amplifying part of *sxy* and its promoter region from *E. coli* K-12 MG1655 using primers PDsxy + sxy253kan-*Kpn*I-r, then digesting fragment as well as vector (pOU253kan) with restriction enzymes *Eco*RI and *Kpn*I. The vector was treated with alkaline phosphatase to inhibit re-ligation. The insert was then ligated into the vector using T4 ligase.

**Plasmid pES2534** was created by amplifying part of *sxy* and its promoter region from *E. coli* K-12 MG1655 using primers PPsxy + sxy253kan-*Kpn*I-r, then digesting fragment as well as vector (pOU253kan) with restriction enzymes *Eco*RI and *Kpn*I. The vector was treated with alkaline phosphatase to inhibit re-ligation. The insert was then ligated into the vector using T4 ligase.

**Plasmid pES2535** was created by amplifying part of *sxy* and its promoter region from *E. coli* K-12 MG1655 using primers DDsxy + sxy253kan-*Kpn*I-r, then digesting fragment as well as vector (pOU253kan) with restriction enzymes *Eco*RI and *Kpn*I. The vector was treated with alkaline phosphatase to inhibit re-ligation. The insert was then ligated into the vector using T4 ligase

**pES91** was constructed in two steps. First the region containing the *rrnB*T1T2 terminators and the gene encoding YFP^unstable^ was PCR amplified using primers *Kpn*IT1T2 (Hunziker et al., 2010) and VENYDN (Mitarai et al., 2015) and plasmid pSEM3131B (Mitarai et al., 2015) as template. The amplified fragment was digested with *Kpn*I and *Bam*HI and inserted into plasmid pLG338 (Stoker et al., 1982) between the same restriction sites (pSEM3034). The *P*_sxy_ promoter region was PCR amplified using primers sxypup and sxypdn and MG1655 chromosomal DNA as template. The fragment was cut with *Eco*RI and *Bam*HI, and inserted between the *Eco*RI and *Bgl*II sites of pSEM3034, located between the *rrnB*T1T2 terminators and the gene encoding YFP.

**Plasmid pES251SxyS26P** was created using site-directed plasmid mutagenesis PCR (Liu and Naismith, 2008) and primers sxyS26Prollingcw + sxyS26Prollingccw using pES251 as template plasmid.

**Plasmid pES251SxyS32G** was created using site-directed plasmid mutagenesis PCR (Liu and Naismith, 2008) and primers sxyS32Grollingcw + sxyS32Grollingccw using pES251 as template plasmid.

**Plasmid pES251SxyD37G** was created using site-directed plasmid mutagenesis PCR (Liu and Naismith, 2008) and primers sxyD37Grollingcw + sxyD37Grollingccw using pES251 as template plasmid.

**Plasmid pES251SxyC73R** was created using site-directed plasmid mutagenesis PCR (Liu and Naismith, 2008) and primers sxyC73Rrollingcw + sxyC73Rrollingccw using pES251 as template plasmid.

**Plasmid pES251SxyS30C and pES251SxyT34S** were found in the screen for compensation of CRPQ194R as described.

**Plasmids pES251_1-122_, pES251S26P_1-122_, pES251S32G_1-122_, pES251D37G_1-122_ and pES251C73R_1-122_** was created by amplifying part of the open reading frame of *sxy* from plasmids pES251, pES251SxyS26P, pES251SxyS32G, pES251SxyD37G and pES251SxyC73R using primers sxycw*Bam*HI and 122SxyNCCW, then digesting fragment as well as vector (pMG25) with restriction enzymes *Bam*HI + *Hind*III. The vector was treated with alkaline phosphatase to inhibit re-ligation. The insert was then ligated into the vector using T4 ligase

**Plasmid pES3221SxyS26P** was created using site-directed plasmid mutagenesis PCR (Liu and Naismith, 2008) and primers sxyS26Prollingcw + sxyS26Prollingccw using pES3221 as template plasmid.

**Plasmid pES3221SxyS32G** was created using site-directed plasmid mutagenesis PCR (Liu and Naismith, 2008) and primers sxyS32Grollingcw + sxyS32Grollingccw using pES3221 as template plasmid.

**Plasmid pES3221SxyD37G** was created using site-directed plasmid mutagenesis PCR (Liu and Naismith, 2008) and primers sxyD37Grollingcw + sxyD37Grollingccw using pES3221 as template plasmid.

**Plasmid pES3221SxyC73R** was created using site-directed plasmid mutagenesis PCR (Liu and Naismith, 2008) and primers sxyC73Rrollingcw + sxyC73Rrollingccw using pES3221 as template plasmid.

**Plasmid pES3221SxyS30C** was created using site-directed plasmid mutagenesis PCR (Liu and Naismith, 2008) and primers sxyS30Crollingcw + sxyS30Crollingccw using pES3221 as template plasmid.

**Plasmids pESCRPQ154LT159A and pES261CRPH22Q** were found in the screen for CRP-S specific CRP mutations created as described.

**Plasmid pES261CRPL196Q** was created using site-directed plasmid mutagenesis PCR (Liu and Naismith, 2008) and primers CRPL196Qrollingcw + CRPL196Qrollingccw using pES261 as template plasmid.

**Plasmid pES261CRPQ194R** was created using site-directed plasmid mutagenesis PCR (Liu and Naismith, 2008) and primers CRPQ194Rrollingcw+ CRPQ194Rrollingccw using pES261 as template plasmid.

**Plasmids pES261CRPL196R/N/S/I/T/P** were created using site-directed plasmid mutagenesis PCR as PCR (Liu and Naismith, 2008) and primer pairs CRPQ196NNNrollingcw + CRPQ196NNNrollingccw using pES261 as template plasmid.

**Plasmids pES261CRPQ194W/K/H/L/E** were created using site-directed plasmid mutagenesis PCR as PCR (Liu and Naismith, 2008) and primer pairs CRPL194NNNrollingcw + CRPL194NNNrollingccw using pES261 as template plasmid.

# Supplementary Figures

## Supplementary Figure S1.


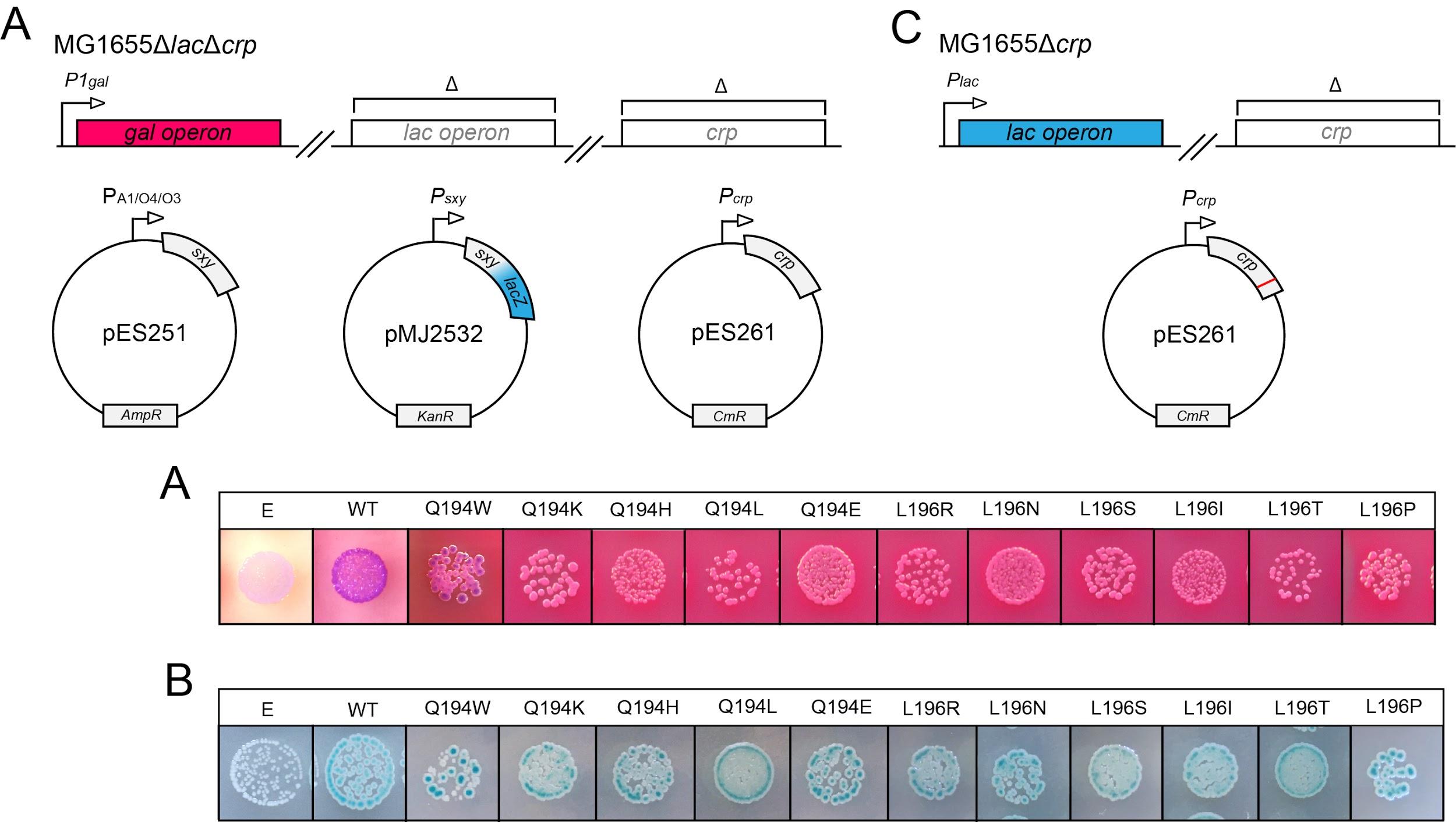


**Figure S1.** Supplemental data to Figure 1.

**A)** Plate showing CRP mutants with different abilities to induce CRP-S sites. MG1655 Δ*lac*Δ*crp* pMJ2532pES251 containing either empty vector E (p15A), pES261, pES261CRPQ194E/H/K/L/W or pES261CRPL196I/N/P/Q/S/T on MacConkey galactose containing X-gal, IPTG and appropriate antibiotics. **B)** Plate showing CRP mutants with different abilities to induce the model Class I CRP dependent promoter *P_lac_*. MG1655Δ*crp* pMJ2532 pES251 containing either empty vector E (p15A), pES261, pES261CRPQ194E/H/K/L/W or pES261CRPL196I/N/P/Q/S/T on LB plates containing X-gal and appropriate antibiotics.

## Supplementary Figure S2

Figure S2. Chromosomal *lac*Z expression in the presence of various CRP mutants.

Quantification of *lac*Z expression in the presence of CRP mutants (see also Figure S1B). Different amino acid substitutions have either no or little effect on the ability of CRP to induce the chromosomal *lac*Z promoter. Cells were grown in M9 medium containing glycerol (0.8 %) and Casamino acids (0.2%) in a shaking incubator at 37 °C. β-galactosidase assays were performed as described previously (Miller JH., 1972). Results shown are the averages of measurements of three biological replicates. Error bars indicate ± 1SEM.

## Supplementary Figure S3.


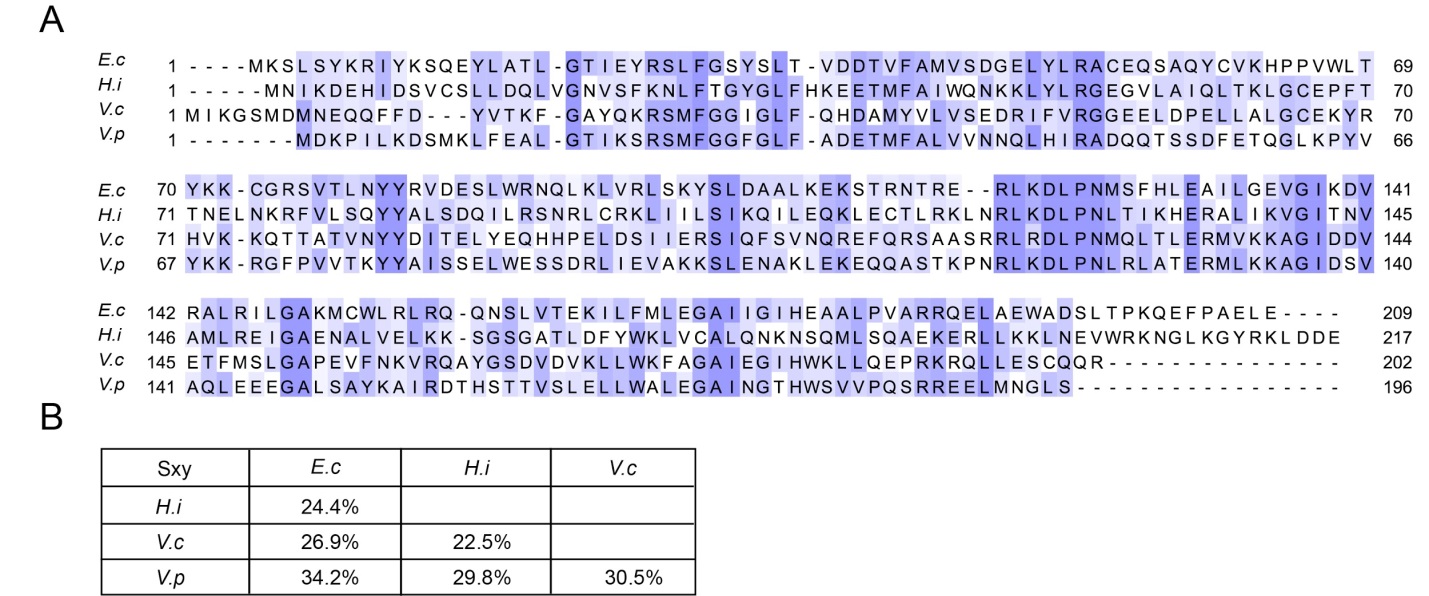


Figure S3. Conservation of Sxy

A) Multiple alignments of Sxy orthologues from *E. coli* (*E.c*), *H.influenzae* (*H.i*), *V. cholerae* (*V.c*) and *V. parahaemolyticus* (*V.p*). Alignment is created using Muscle (Edgar, 2004), visualized in Jalview and colored according to the Blossum62 scoring matrix color scheme (Waterhouse et al., 2009). Increasing color intensity corresponds to the level of conservation at the position. B) Sequence identity matrix based on pairwise alignment of Sxy orthologous shown in A.

# Supplementary Tables

## Supplementary Table S1.

| **Table S1. Strains** | **Genotype** | **Reference** |
| --- | --- | --- |
| **MG1655∆*lac*** | MG1655∆*lacIZYA* | Laboratory collection |
| **MG1655∆*lac*Δ*cyaA*** | MG1655∆*lacIZYA*∆*cyaA* (MJ10) | (Jaskolska and Gerdes, 2015) |
| **MG1655∆*crp*** | MG1655∆*crp*-96 zhd-732::tn10 | Laboratory collection (Milena Jaskolska, unpublished) |
| **MG1655∆*lac*Δ*crp*** | MG1655∆*lacIZYA*∆*crp*-96 zhd-732::Tn10 (MJ11) | (Jaskolska and Gerdes, 2015) |
| **MG1655** | *E. coli* K-12 F^–^ λ^–^ *ilvG*^–^ *rfb-50 rph-1* | Laboratory collection |
| **ES10** | MG1655∆*lacIZYA* SxyS32G | This study |

## Supplementary Table S2.

| **Table S2. plasmids** | **Relevant details** | **Reference** |
| --- | --- | --- |
| **pOU253kan** | mini-R1, *lacZYA* translational fusion  vector, Kan^R^ | Laboratory collection |
| **pMJ2532** | pOU253kan::*sxy*P, translational sxy-lacZ fusion | (Jaskolska and Gerdes, 2015) |
| **pES2533** | pMJ2532 PD | This study |
| **pES2534** | pMJ2532 PP | This study |
| **pES2535** | pMJ2532 DD | This study |
| **pMG25** | pUC *lacI^q^* P_A1/O4/O3_ Amp^R^ | Laboratory collection |
| **pES251** | pMG25::*sxy* | This study |
| **pES251SxyS26P** | pES251 SxyS26P | This study |
| **pES251SxyS30C** | pES251 SxyS30C | This study |
| **pES251SxyS32G** | pES251 SxyS32G | This study |
| **pES251SxyT34S** | pES251 SxyT34S | This study |
| **pES251SxyD37G** | pES251 SxyD37G | This study |
| **pES251SxyC73R** | pES251 SxyC73R | This study |
| **pES251Sxy**_1-122_ | pMG25::*sxy*_1-122_ | This study |
| **pES251SxyS26P**_1-122_ | pES251Sxy_1-122_ SxyS26P | This study |
| **pES251SxyS32G**_1-122_ | pES251Sxy_1-122_ SxyS32G | This study |
| **pES251SxyD37G**_1-122_ | pES251Sxy_1-122_ SxyD37G | This study |
| **pES251SxyC73R**_1-122_ | pES251Sxy_1-122_ SxyC73R | This study |
| **pBR322** | pMB1 Amp^R^ Tet^R^ | Laboratory collection |
| **pES3221** | pBR322::P_sxy_*sxy* | This study |
| **pES3221SxyS26P** | pES3221 SxyS26P | This study |
| **pES3221SxyS30C** | pES3221 SxyS30C | This study |
| **pES3221SxyS32G** | pES3221 SxyS32G | This study |
| **pES3221SxyD37G** | pES3221 SxyD37G | This study |
| **pES3221SxyC73R** | pES3221 SxyC73R | This study |
| **p15A** | p15A | Addgene plasmid #44249 (Qi et al., 2013) |
| **pES261** | p15A, *P_crp_-crp* | This study |
| **pES261CRPQ194R** | pES261 CRPQ194R | This study |
| **pES261CRPL196Q** | pES261 CRPL196Q | This study |
| **pES261CRPQ154L,T159A** | pES261 CRPQ154L,T159A | This study |
| **pES261CRPH22Q** | pES261CRPH22Q | This study |
| **pLG338** | KanR | (Stoker et al., 1982) |
| **pES91** | pLG338 based reporter, P*_sxy_*-*yfp* fusion | This study |
| **pES261CRPL196N** | pES261CRPL196N | This study |
| **pES261CRPL196S** | pES261CRPL196S | This study |
| **pES261CRPL196I** | pES261CRPL196I | This study |
| **pES261CRPL196T** | pES261CRPL196T | This study |
| **pES261CRPL196R** | pES261CRPL196R | This study |
| **pES261CRPL196P** | pES261CRPL196P | This study |
| **pES261CRPQ194L** | pES261CRPQ194L | This study |
| **pES261CRPQ194K** | pES261CRPQ194K | This study |
| **pES261CRPQ194H** | pES261CRPQ194H | This study |
| **pES261CRPQ194W** | pES261CRPQ194W | This study |
| **pES261CRPQ194E** | pES261CRPQ194E | This study |
| **pES261CRPQ194I** | pES261CRPQ194I | This study |

## Supplementary Table S3.

| **Table S3. Oligonucleotides** |  |
| --- | --- |
| **sxypwrg100cw** | 5’CACAAGAATACCTGGCAACGTTGGGCACAATTGAATACCG CGCCTTACGCCCCGCCCTGC |
| **sxypwrg100ccw** | 5´GCCCGAAGATACAACTCACCATCAGAAACCATCGCAAACAC CTAGACTATATTACCCTGTT |
| **sxymutrekfragcw** | 5´ CACAAGAATACCTGGCAACG |
| **sxymutrekfragccw** | 5´ GCCCGAAGATACAACTCACC |
| **500upsxycw** | 5´ AAAAGAATTCGAGCTGGCTAATCTGCATTACTTTCGT |
| **sxyccw*hin*dIII** | 5´CCCCCAAGCTTTTACTCAAGTTCCGCAGGAAACTCCTG |
| **sxyD37Grollingcw** | 5´CGTTGACGGCACGGTGTTTGCGATG |
| **sxyD37Grollingccw** | 5´ACACCGTGCCGTCAACGGTCAGGCT |
| **sxyC73Rrollingcw** | 5´GCTGACATATAAAAAGCGTGGCCGATCCGTTAC |
| **sxyC73Rrollingccw** | 5´TCGGCCACGCTTTTTATATGTCAGCCAGACAGG |
| **sxyS32Grollingcw** | 5´GTTTGGCAGTTACGGCCTGACCGTTGACGA |
| **sxyS32Grollingccw** | 5´GGTCAGGCCGTAACTGCCAAACAATGATCG |
| **sxyS26Prollingcw** | 5´TTGAATACCGACCATTGTTTGGCAGTTACAGC |
| **sxyS26Prollingccw** | 5´TGCCAAACAATGGTCGGTATTCAATTGTGCCC |
| **sxyS30Crollingcw** | 5´TCATTGTTTGGCTGTTACAGCCTGACCGTTGA |
| **sxyS30Crollingccw** | 5´CAGGCTGTAACAGCCAAACAATGATCGGTATT |
| **sxycw*Bam*HI** | 5´ CCCCCGGATCCAAAATAAGGAGGAAAAAAAA ATGAAAAGCC TCTCCTATAAG |
| **500upCRPcw** | 5´ CCCCCAGATCTCACAATCGACCACATCCTGAC |
| **CRP*Xho*ICCW** | 5’CCCCCCTCGAGTTAACGAGTGCCGTAAACGACG |
| **ORFsxyCW** | 5' CCCCC GGATCCGTTAATGTGATCATTCTTTTATG |
| **ORFCRPCW** | 5' CCCCC AAGCTTATAACAGAGGATAACCGCGC |
| **CRPL196Qrollingcw** | 5’ AAGATCAGAACCAGATCTCCGCACACGGTA |
| **CRPL196Qrollingccw** | 5’ GCGGAGATCTGGTTCTGATCTTCCAGCATC |
| **CRPQ194Rrollingcw** | 5’ ATGCTGGAAGATCGGAACCTGATCTCCGCACA |
| **CRPQ194Rrollingccw** | 5’ GATCAGGTTCCGATCTTCCAGCATCTTCAGAA |
| **CRPQ196NNNrollingcw** | 5’ TGGAAGATCAGAACNNNATCTCCGCACACGGTAAA |
| **CRPQ196NNNrollingccw** | 5’ GTGCGGAGATNNNGTTCTGATCTTCCAGCATCTTC |
| **CRPL194NNNrollingcw** | 5´ AGATGCTGGAAGATNNNAACCTGATCTCCGCACAC |
| **CRPL194NNNrollingccw** | 5´ AGATCAGGTTNNNATCTTCCAGCATCTTCAGAATG |
| **DDsxy** | 5’CCCCCGAATTCACATCCAGTGACAACAAAGATCAACCCTATTTTCGGAAAGAGCCTTCCGAATTTTGTCGTTGGTGACGGG |
| **PPsxy** | 5’CCCCCGAATTCACATCCAGTGACAACAAAGATCAACCCTATTTTTGCGAAGAGCCTCGCAAATTTTGTCGTTGGTGACGGG |
| **PDsxy** | 5’CCCCCGAATTCACATCCAGTGACAACAAAGATCAACCCTATTTTTGCGAAGAGCCTTCCGAATTTTGTCGTTGGTGACGGG |
| **sxy253kan-*Kpn*I-r** | 5´CCCCCGGTACCAAACACCGTGTCGTC |
| **Sxypup** | 5’ AAAAGAATTCGAGCTGGCTAATCTGCATTACTTTCGT |
| **Sxypdn** | 5’ AAAAGGATCCTTGTGATTTATAGATCCGCTTAT |
| **122SxyNCCW** | 5’ CCCCCAAGCTTCTACAAATCTTTCAGTCTTTCCC |

# Supplementary References

Blank, K., Hensel, M., and Gerlach, R.G. (2011). Rapid and highly efficient method for scarless mutagenesis within the *Salmonella enterica* chromosome. *PLoS One 6*, e15763.

Edgar, R.C. (2004). MUSCLE: multiple sequence alignment with high accuracy and high throughput. *Nucleic Acids Res 32*, 1792-1797.

Hunziker, A., Tuboly, C., Horvath, P., Krishna, S., and Semsey, S. (2010). Genetic flexibility of regulatory networks. *Proc Natl Acad Sci U S A 107*, 12998-13003.

Jaskolska, M., and Gerdes, K. (2015). CRP-dependent positive autoregulation and proteolytic degradation regulate competence activator Sxy of *Escherichia coli. Mol Microbiol 95*, 833-845.

Kelley, L.A., Mezulis, S., Yates, C.M., Wass, M.N., and Sternberg, M.J.E. (2015). The Phyre2 web portal for protein modeling, prediction and analysis. *Nature Protocols 10*, 845.

Liu, H., and Naismith, J.H. (2008). An efficient one-step site-directed deletion, insertion, single and multiple-site plasmid mutagenesis protocol. *BMC Biotechnol 8*, 91.

Miller, J. H. (1972). Experiments in molecular genetics, Cold Spring Harbor Laboratory, Cold Spring Harbor, New York. p. 352-355.

Mitarai, N., Jensen, M.H., and Semsey, S. (2015). Coupled positive and negative feedbacks produce diverse gene expression patterns in colonies. *MBio 6*.

Qi, L.S., Larson, M.H., Gilbert, L.A., Doudna, J.A., Weissman, J.S., Arkin, A.P., and Lim, W.A. (2013). Repurposing CRISPR as an RNA-guided platform for sequence-specific control of gene expression. *Cell 152*, 1173-1183.

Stoker, N.G., Fairweather, N.F., and Spratt, B.G. (1982). Versatile low-copy-number plasmid vectors for cloning in *Escherichia coli*. *Gene 18*, 335-341.

Waterhouse, A.M., Procter, J.B., Martin, D.M., Clamp, M., and Barton, G.J. (2009). Jalview Version 2--a multiple sequence alignment editor and analysis workbench. *Bioinformatics 25*, 1189-1191.
